# The cerebellum is associated with 2-year prognosis in patients with high-frequency migraine

**DOI:** 10.1186/s10194-020-01096-4

**Published:** 2020-03-18

**Authors:** Hung-Yu Liu, Pei-Lin Lee, Kun-Hsien Chou, Kuan-Lin Lai, Yen-Feng Wang, Shih-Pin Chen, Wei-Ta Chen, Shuu-Jiun Wang

**Affiliations:** 1 Department of Neurology, Neurological Institute, Taipei Veterans General Hospital, No. 201, Sec. 2 Shih-Pai Rd, Taipei, Taiwan; 2 School of Medicine, National Yang-Ming University, Taipei, Taiwan; 3 Institute of Neuroscience, National Yang-Ming University, Taipei, Taiwan; 4 Brain Research Center, National Yang-Ming University, Taipei, Taiwan; 5 Division of Translational Research, Department of Medical Research, Taipei Veterans General Hospital, Taipei, Taiwan

**Keywords:** Cerebellum, Migraine, High frequency, Outcome, MRI

## Abstract

**Background:**

The increase of headache frequency is associated with higher headache related disability and lower quality of life in patients with migraine. However, the pathophysiology of migraine progression, persistence, or remission is elusive. The purpose of this study is to identify the brain signatures that are predictive of the long-term outcomes among patients with high-frequency migraine (HFM: 10–30 headache days/month).

**Methods:**

We prospectively enrolled patients with HFM and healthy controls and collected their baseline clinical profiles and brain-MRI data at first visit. We longitudinally followed the patients and determined their outcomes at 2-year follow-up. Good outcome was defined as ≥50% reduction of baseline headache days and poor outcome was defined as reduction < 50% or frequency increase. Voxel-based morphometry was used to study gray matter volume (GMV), and structural covariance was used to investigate structural connectivity.

**Results:**

Among 56 patients with HFM, 37 had good outcome and 19 poor outcome. Compared to the healthy controls (*n* = 37), patients with poor outcome had decreased GMV over the left posterior cingulate gyrus, and increased GMV over the bilateral cerebellum and the right precentral gyrus. Further, patients with poor outcome had greater GMV over the right and the left cerebella compared to patients with good outcome, and the GMVs of the cerebella were correlated to 2-year headache frequencies (right: r = 0.38, *P* = 0.005; left: r = 0.35, *P* = 0.009). Structural connectivity were increased between the cerebellum and the cuneus, the calcarine cortex, and the temporal lobe, respectively, in patients with poor outcome, and was decreased between the cerebellum and the prefrontal cortex in patients with poor outcome. The structural covariance integrities between the right cerebellum and the right cuneus were correlated to 2-year headache frequencies (r = 0.36, *P* = 0.008).

**Conclusions:**

Structural volume and connectivity changes of the cerebellum may underlie headache persistence in patients with HFM.

## Introduction

Migraine is a recurrent headache disorder, with its attack frequency fluctuating during the disease course [1–3]. Some patients with episodic migraine may transform to chronic migraine (CM: headache days ≥15/month and migraine days ≥8/month for more than 3 months). Fortunately, most of the patients with CM have headache remission to episodic form with time. However, some patients with CM persistently suffer from frequent migraine attacks, with their headache-related burdens influencing their occupations, as well as their social and family functioning [4].

The pathophysiology of migraine progression, persistence, or remission is yet elusive. Current evidences have shown some risk factors for migraine progression or persistence. Baseline high-frequency headaches, medication overuse, and depression are associated with migraine progression [5, 6], while medication overuse and chronic musculoskeletal complaints are predictors of chronic headache persistence [2]. In the field of neuroimage, altered brain volumes and activities have been demonstrated in patients with CM compared to the patients with episodic migraine or the healthy controls (HC). However, whether such changes reflect the current status of frequent attacks, the longitudinal consequences of migraine attacks, or are pathogenic and involved in headache chronification is not clearly understood [7–9].

This study aims to explore the neuroimage signatures related to long-term prognosis in patients with migraine with frequent attacks. High-frequency episodic migraine (10–14 headache days/month) is the most susceptible group among episodic migraine to evolve to CM, and is as disabling as CM [10–12]. Therefore, we considered high-frequency episodic migraine in a continuum with CM, and investigated both types as a single entity, termed “high-frequency migraine” (HFM; 10–30 headache days/month). We prospectively recruited patients with HFM, obtained their clinical profiles and brain-MRI at baseline, and longitudinally followed-up their headache status after 2 years. The outcomes of the patients were determined by their follow-up 2-year headache frequency. We explored the clinical and neuroimaging data between patients with HFM with different outcomes. We sought to identify the brain structures that are pathogenic to chronic headache with frequent attacks.

## Methods

### Participants

Headache specialists at the Headache Clinic of the Taipei Veterans General Hospital prospectively surveyed patients aged 20–60 years with newly-diagnosed migraine from May 2011 to Jan 2017. The diagnosis of migraine followed the International Classification of Headache Disorders-3 criteria [13]. We included patients with high frequency episodic migraine and CM, and excluded patients with severe depression, i.e., Hospital Anxiety and Depression Scale-Depression (HADS-D) score ≥ 15, or those with comorbid medication overuse, i.e., using abortive headache medication for more than 10 or 15 days per month [14]. We recruited age- and sex-matched HC for comparison.

All participants were right-handed, denied any history of systemic or major neurological diseases, and presented with normal neurologic examinations. We encrypted information that could potentially expose individual identity.

### Standard protocol approvals, registrations, and patient consents

All participants completed informed consent forms after receiving a complete explanation of the study. The Institutional Review Board of our hospital approved the study protocol.

### Study design

All participants filled out a semi-structured questionnaire at their first visit to obtain demographic information and headache profiles. We defined headache frequency as the average number of headache days per month in the last 3 months and duration of headache history as the duration in years between the first migraine episode and the date of the first visit to our headache clinic. We evaluated mood based on severity of depression and anxiety using the HADS [14] and functional disability caused by migraine using the Migraine Disability Assessment Scale (MIDAS) [15].

Each participant underwent scheduled MRI during the interictal period, defined as absence of acute migraine attack within 2 days prior and subsequent to the date of image acquisition. We rescheduled the scanning session if there was an acute migraine episode during this period or use of analgesics, triptans, or ergots for any reason within 48 h prior to scanning.

### Follow-up

After the MRI scanning, all included patients were treated at our headache clinic by headache specialists according to their clinical experience. Two years after the first visit, physician interviewed the patients by telephone to assess their migraine status and headache frequency within the last 3 months. Based on the comparison of current headache frequency to that at first visit, we defined good outcome as ≥50% reduction in baseline headache frequency and poor outcome as lower than 50% reduction in headache frequency or as frequency increase. Medical records were also reviewed to assess the use of migraine medication in these patients. Among the 56 patients included in our analysis, 48 were treated with both migraine prophylactic (including topiramate, propranolol, metoprolol, flunarizine, valproic acid, tricyclic acid, or amitriptyline) and abortive medications (sumatriptan or non- steroidal anti-inflammatory drugs), while the other 8 patients were treated with abortive medications only.

### MRI data acquisition

We used the same 3.0 T GE Discovery MR750 scanner (General Electric Healthcare, Milwaukee, WI, USA) at Taipei Veterans General Hospital to acquire all data with a standard eight-channel phase array head coil.

We acquired the T1-weighted anatomical scans with two different acquisition pulse sequences including: 1) an inversion recovery prepared fast spoiled gradient-recalled echo sequence (IR-FSPGR) and 2) an IR-FSPGR-brain volume imaging (BRAVO) sequence.

Of note, 32 of 56 patients with HFM and 29 of 37 HC in this study were also included in a dataset that was recently used to analyze hippocampal volume changes in patients with migraine [16]; these patients underwent the first acquisition pulse sequence, whereas the rest and the HC underwent the second acquisition pulse sequence.

The detailed imaging parameters of each protocol were as follows: repetition time/echo time/inversion time = 9.4 (IR-FSPGR sequence) or 9.2 (IR-FSPGR-BRAVO sequence) / 4.0 (IR-FSPGR sequence) or 3.7 (IR-FSPGR-BRAVO sequence) / 450 ms; flip angle = 12°, matrix size = 256 × 256, field of view = 256 × 256 mm^2^, number of excitations = 1, slice thickness = 1 mm without inter-slice gap and interpolation, and 172 (IR-FSPGR sequence) or 168 (IR-FSPGR-BRAVO sequence) axial contiguous slices. An experienced neuroradiologist visually inspected all MRI scans to exclude any organic brain disorders; no participant was excluded for brain abnormalities. Before subsequent image processing, we reoriented all T1-weighed scans using a center-of-mass approach to minimize the position difference during the data acquisition.

### Calculation of gray matter volume information

To estimate individual voxel-wise gray matter volume (GMV) maps, we processed T1-weighted scans using the voxel based morphometry (VBM) [17] pipeline with Statistical Parametric Mapping 12 (SPM12, version 7487, Wellcome Institute of Neurology, University College London, UK) under the MATLAB environment (version R2015b; Mathworks, Natick, MA). We corrected individual T1-weighted scans for intensity inhomogeneities, segmented them into GM, white matter (WM), and cerebrospinal fluid (CSF); and initially rigid-aligned them to the Montreal Neurological Institute (MNI) space using the SPM12 “Segment” function. To improve tissue classification accuracy of the subcortical areas, which are highly involved in the pathophysiology of migraine, we incorporated the enhanced tissue probability maps for subcortical regions to the above tissue segmentation procedure [18]. We then used the Diffeomorphic Anatomical Registration through Exponentiated Lie algebra (DARTEL) toolbox to generate study-specific tissue templates, by iteratively registering the ridge-aligned GM and WM segments of all study participants, and to further warp individual tissue segments to the constructed templates [19]. We modulated the individual MNI-space GM tissue segments with the corresponding DARTEL flow field to ensure that the following statistical analyses would be more sensitive to local GMV changes. Finally, we smoothed the modulated GMV maps with an isotropic Gaussian filter (full width at half maximum = 8 mm) and we further excluded voxels with GM probability lower than 0.2. We set the final spatial resolution of all GMV maps to 1.5 mm^3^. We estimated the individual global tissue volume and total intracranial volume (TIV = GM + WM + CSF volumes) in native T1 space to adjust for the effect of global brain size in the following statistical analyses.

### Minimization of the influence of different acquisition protocols using data harmonization modeling

In this study, we used the ComBat harmonization approach to reduce the potential influence of different image acquisition protocols in GMV measurements [20]. ComBat was originally designed to correct for “batch effects” in genomic studies, and recent multi-site neuroimaging studies adapted this approach to remove unwanted non-biological variability while preserving meaningful associations between image variables and covariate of interests [21–23]. This approach uses a multivariate linear mixed effects regression with terms for biological variables and imaging protocols to model quantitative measurements (for example: voxel-wise GMV maps in the current study). In more detail, we included “group” as a covariate of interest to preserve potential biological trends in the data and simultaneously corrected for the effect of different acquisition protocols.

### Statistical analysis

#### Analyses of demographic and clinical data

The descriptive data in the demographic and clinical profiles are presented as mean ± standard deviation or numbers and percentages. We used the chi-square test to test for differences in categorical data. We used Student’s t test to compare the means of normally-distributed continuous variables, and the Mann–Whitney U test to compare non-normally-distributed variables, i.e., the MIDAS scores.

#### Analyses of voxel-wise imaging-based investigations

We used SPM12 with appropriate statistical models to perform the following voxel-wise statistical analyses. We adopted the cluster-extent statistical approach with the updated “3dFWHMx” and “3dClustSim” functions (available in the Analysis of Functional Neuroimages software, version 19.1.18; 10,000 Monte Carlo simulations with explicit GM mask) to correct for multiple comparisons across the whole-brain voxels. An initial voxel-level *P*-value < 0.005 with 257 extended voxels was considered statistically significant at cluster-level family-wise error (FWE) rate-corrected P-value < 0.05 for all voxel-wise statistical analyses (including VBM and structural covariance (SC) network analysis). The details of the statistical models are listed below.

### Identification of GMV alterations among patients with HFM with different outcomes and HC

To identify the GMV difference among patients with HFM with different outcome status and the HC, a single-factor three-level (HFM with good outcome, HFM with poor outcome, and HC) analysis of covariance (ANCOVA) design was employed, with age, sex, TIV, and HADS scores entered as nuisance variables. The following contrasts were tested: HFM vs. HC, HFM with good outcome vs. HC, HFM with poor outcome vs. HC, and HFM with good outcome vs. HFM with poor outcome. We extracted, averaged, and correlated the GMV of the clusters with a significant between-group effect with headache profiles using partial Pearson’s correlation to investigate the clinical relevance. We entered age, sex, TIV, and HADS scores as nuisance variables in the correlation analysis.

### Exploration of the changes in the SC network in patients with different outcomes

SC is a volumetric correlation measurement between two brain regions. SC network analysis was recently proposed as a surrogate approach to characterizing structural connectivity profiles between distinct anatomic brain regions [24]. We generated SC networks using hypothesis-driven, seed-based correlation analysis [25] or data-driven, independent component analysis. SC networks reflect the shared covariance of brain morphologic features across the study participants and provide a quantitative means to studying cortical morphometric organization. In addition, the topology of the SC network is highly concordant with gene expression patterns and recapitulates intrinsic functional network architecture [26, 27].

We used three cerebellar regions with significant GMV alterations between the outcome groups as predefined regions of interest (ROIs) for the SC analyses. We constructed three multiple regression models for the respective ROIs to explore potential differences in the SC network between the outcome groups [28]. We integrated a group main effect term (good vs. poor outcome), a mean ROI volume main effect, and a group x mean ROI volume interaction term into the statistical model and included age, sex, TIV, and HADS scores to adjust for potential nuisance effects. The changes in SC integrity of the predefined ROI with identified clusters between the outcome groups could be identified by evaluating the statistical significance of the corresponding interaction term of the constructed models.

#### Investigation of SC integrity and long-term headache frequencies

To investigate if the changes in SC integrity between the predefined ROIs and identified clusters were correlated to 2-year headache frequencies, we applied a recently-proposed approach to obtain a single measure that could quantify the integrity of SC for each individual [29]. We correlated the SC measures for each patient with their 2-year headache frequencies using partial Pearson’s correlation after controlling for age, sex, and HADS scores.

#### Predictive values of clinical profiles and neuroimaging data for headache outcomes

We constructed two logistic regression models to assess the predictive values of clinical profiles only and clinical profiles with neuroimaging results for headache outcomes, respectively. We estimated the predictive values of the regression models using the area under the receiver operating characteristic curve (AUC).

We performed all statistical analyses using SPSS version 21.0 for Windows (IBM Corp., Armonk, NY), and a *P* value < 0.05 was regarded as significant.

## Results

### Demographics, clinical profiles, and headache outcomes

We enrolled 64 patients with HFM and studied their clinical headache profiles and neuroimaging data. Eight patients (12.5%) were excluded from the analysis because they were either lost to follow-up after 2 years or could not clearly recall their current 3-month headache frequencies and provide accurate information regarding their headache days, rendering categorization of their headache outcomes difficult. We included the data of the remaining 56 patients with HFM (44 were CM and 12 were high frequency episodic migraine) in the final analyses. Of note, the 44 patients with CM and the 12 patients with high frequency episodic migraine did not differ in demographics, headache features, impacts, and outcome, except for a higher score of HADS-D and baseline headache frequency in those with CM (Supplement Table 1).

Patients with HFM were age- and sex-matched to the 37 HC, but they had higher HADS-A and HADS-D scores compared to the HC. Among patients with HFM, 37 had good outcome, whereas the other 19 had poor outcome. Patients with different outcomes were similar in age and sex, HADS scores, and headache characteristics, but patients with poor outcome had a borderline higher MIDAS score than did those with good outcome (*P* = 0.050). Table 1 presents the demographics and clinical profiles.

**Table 1:** Demographics, clinical profiles, and neuroimaging data of patients with HFM and HC

	HC (*n* = 37)	HFM (*n* = 56)	HFM Good outcome (*n* = 37)	HFM Poor outcome (*n* = 19)
Age	39.4 ± 9.3	40.3 ± 10.5	40.8 ± 11.1	39.4 ± 9.6
Sex (F/M)	27/10	43/13	27/10	16/3
Aura (%)	nil	9 (16%)	7 (18.9%)	2 (10.5%)
Headache frequency-baseline (d/m)	nil	19.2 ± 7.1	18.9 ± 7.1	19.7 ± 7.4
Chronic migraine	nil	44 (78.6%)	29 (78.4%)	15 (78.9%)
Disease duration (years)	nil	17.2 ± 11.3	15.7 ± 10.2	20.1 ± 13.0
Headache intensity (NRS 0–10)	nil	6.5 ± 1.7	6.6 ± 1.7	6.3 ± 2.0
MIDAS	nil	43.9 ± 49.0	20 ± 50	62.5 ± 92
HADS-A	4.7 ± 2.5^#^	8.9 ± 4.3	9.1 ± 4.3	8.5 ± 4.3
HADS-D	3.5 ± 2.8^#^	6.8 ± 5.0	6.7 ± 5.5	6.8 ± 4.2
Prophylactic drugs use	nil	48 (85.7%)	30 (81.1%)	18 (94.7%)
Prophylactic drugs use at 2-year follow-up	nil	16 (28.6%)	10 (27%)	6 (31.6%)
Headache frequency at 2-year follow-up (d/m)	nil	9.0 ± 9.5	3.3 ± 2.4*	20.3 ± 7.8
TIV (mm^3^)	1430.8 ± 129.6	1399.8 ± 132.5	1404.7 ± 124.6	1390.2 ± 149.7

*HC* healthy controls, *HFM* high frequency migraine, *NRS* numeric rating scale, *MIDAS* Migraine Disability Assessment, *HADS-A* the anxiety subscale of the Hospital Anxiety and Depression Scale, *HADS-D* the depression subscale of the Hospital Anxiety and Depression Scale, *TIV* total intracranial volume

^#^ denotes difference comparing patients with HFM to HC, *p* < 0.05

*denotes difference comparing patients with HFM with good to poor outcomes, *p* < 0.05

### GMV difference between patients with HFM and HC

**Fig. 1:**
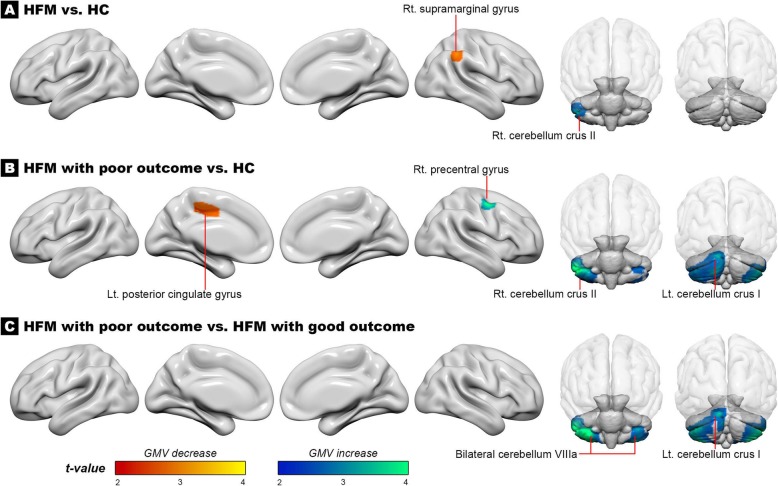
The red and blue markers are regions of different gray matter volume (**a**) between patients with high-frequency migraine (HFM) and healthy controls (HC); (**b**) between patients with poor outcome and HC; (**c**) between patients with different outcomes

Compared to HC, patients with HFM had decreased GMV over the right supramarginal gyrus and increased GMV over the right cerebellar crus II (Table 2 and Fig. 1a). The volume of the right cerebellar crus II was correlated to scores of MIDAS in patients with HFM (*r* = 0.41, *p* = 0.004). Otherwise, the two structures were not correlated to the other headache profiles in patients with HFM. There was no significant difference in GMV between patients with good outcome and HC. However, patients with poor outcome had decreased GMV over the left posterior cingulate gyrus and increased GMV over the bilateral cerebellum and the right precentral gyrus compared to HC (Table 2 and Fig. 1b).

**Table 2:** Difference in GMV among patients with HFM with different outcomes and HC

MNI coordinates			Voxel size	Anatomical region	Local peak *T*-value
x	y	z			
***HC > HFM***					
50	−45	41	266	Right Supramarginal Gyrus	3.41
***HC < HFM***					
45	−46	−43	407	Right Cerebellum Crus II	3.45
***HC > HFM with poor outcome***					
-14	−20	38	287	Left Posterior Cingulate Gyrus	3.00
***HC < HFM with poor outcome***					
47	−47	−44	4408	Right Cerebellum Crus II	4.69
45	−5	56	262	Right Precentral Gyrus	3.66
-11	−84	−26	3285	Left Cerebellum Crus I	3.63
***Good outcome < Poor outcome***					
33	−43	−49	6136	Right Cerebellum VIIIa	4.82
-32	− 53	− 54	3938	Left Cerebellum VIIIa	4.08
-8	−84	−23	560	Left Cerebellum Crus I	3.63

*GMV* gray matter volume, *HC* healthy controls, *HFM* high frequency migraine

### GMV difference between patients with HFM with poor and good outcomes

Compared to patients with good outcome, those with poor outcome had increased GMV over the right cerebellar VIIIa, the left cerebellar VIIIa, and the left cerebellum crus I (Table 2 and Fig. 1c). This finding did not change after further controlling for disease duration or MIDAS score.

The volume of the right cerebellar VIIIa was correlated to disease duration (*r* = 0.34, *P* = 0.014). In addition, the volumes of the right and left cerebellar VIIIa areas were correlated to 2-year headache frequencies (*r* = 0.38, *P* = 0.005; *r* = 0.35, *P* = 0.009, respectively), and the correlation persisted over the right cerebellar VIIIa (*r* = 0.31, *P* = 0.032) after further controlling for disease duration and MIDAS score.

Due to the arbitrary definition of ≥10 days/month in HFM, we also tested the difference in GMV between the two outcome groups among patients with CM only. The results were similar in that patients with CM with a poor outcome had increased GMV over the right and the left cerebella (Supplement Table 2).

### SC networks between the three cerebellar regions and other brain regions in patients with different outcomes

**Fig. 2:**
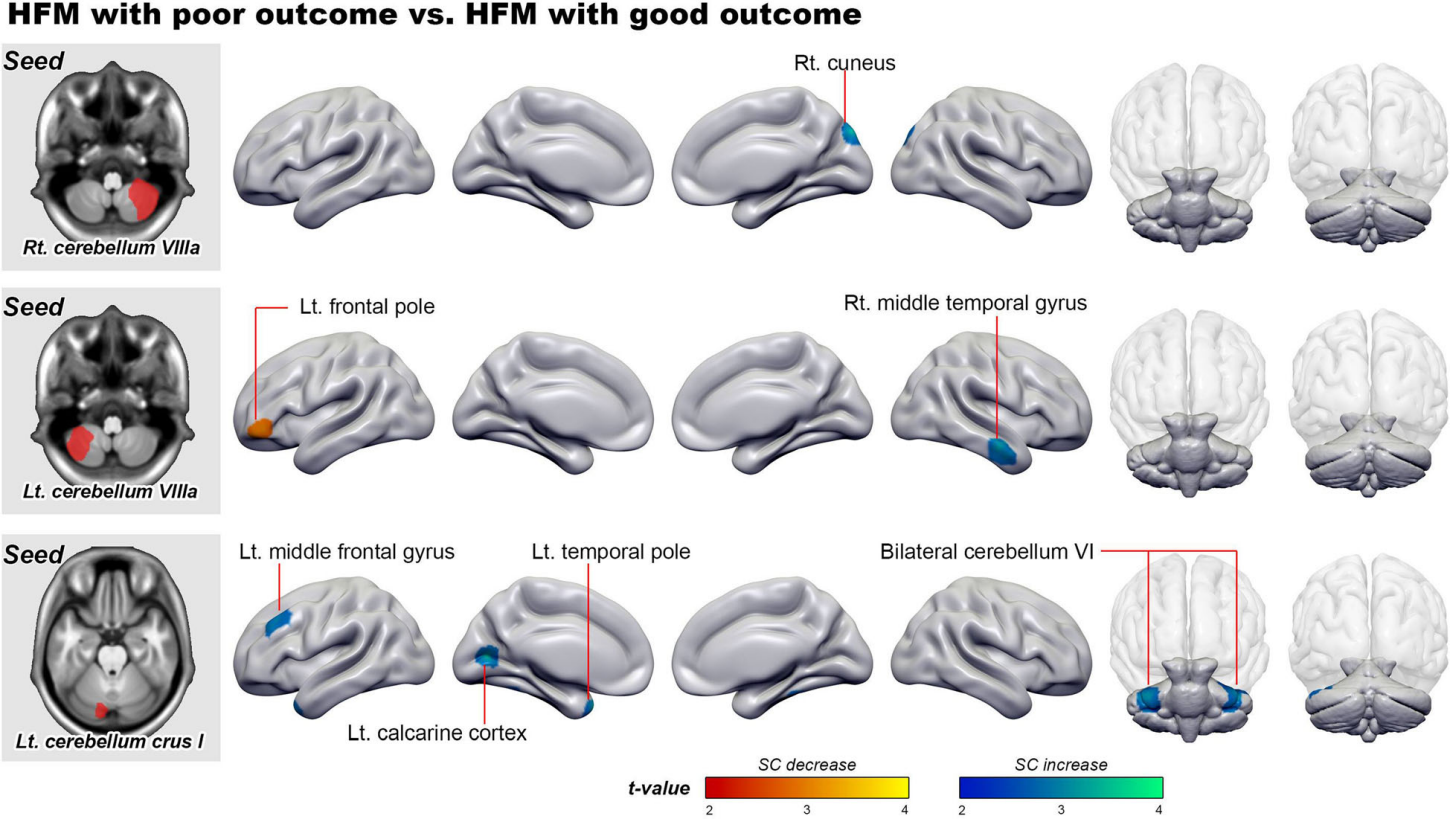
Three cerebellar seeds and the changes in the structural covariance (SC) network from the three seeds to the whole brain between patients with different outcomes. The blue and red markers are regions that displayed different SC with the seeds between the outcome groups

We tested the interactive effect of outcome on the SC networks between the three cerebellar regions that showed volumetric difference between outcome groups and the other regions of the whole brain. In patients with poor outcome, the right cerebellar VIIIa showed an increase of SC with the right cuneus, the left cerebellar VIIIa showed an increase of SC with the right temporal gyrus, and the left cerebellum crus I showed increases of SC with the left temporal pole, left middle frontal gyrus, left calcarine cortex, and left and right cerebellum VI areas. Conversely, the SC between the left cerebellar VIIIa and the left frontal pole was decreased in patients with poor outcome (Table 3 and Fig. 2).

**Table 3:** Difference in structural covariance between patients with HFM with different outcomes

MNI coordinates			Voxel size	Anatomical region	Local peak *T*-value
x	y	z			
**Seed 1: Right cerebellum VIIIa**					
***Poor outcome > Good outcome***					
14	−84	38	272	Right cuneus	3.53
**Seed 2: Left cerebellum VIIIa**					
***Poor outcome > Good outcome***					
56	−13	−23	325	Right middle temporal gyrus	3.64
***Poor outcome < Good outcome***					
-48	47	−6	331	Left frontal pole	3.65
**Seed 3: Left cerebellum crus I**					
***Poor outcome > Good outcome***					
-38	23	−39	557	Left temporal pole	4.30
-30	29	50	420	Left middle frontal gyrus	3.49
-12	−72	9	292	Left calcarine cortex	3.49
-39	−45	−30	918	Left cerebellum VI	3.40
37	−41	−32	686	Right cerebellum VI	3.29

*HFM* high frequency migraine

Further, the SC integrities between the right cerebellar VIIIa and right cuneus were correlated to 2-year headache frequencies (*r* = 0.36, *P* = 0.008).

### Predictive values of cerebellar GMV and its SC integrity for headache outcomes

In the logistic regression analysis, the GMV of the right cerebellar VIIIa and the integrity of the SC between the right cerebellar VIIIa with the right cuneus were independent predictors of poor headache outcome (*P* = 0.006 and *P* = 0.005, respectively). When considering only the clinical profiles (age, sex, disease duration, baseline headache frequency, and HADS and MIDAS scores), the regression model could distinguish patients with poor outcomes from those with good outcomes with fair discrimination (AUC = 0.72). When we added the GMV data of the right cerebellar VIIIa and of the integrity of its SC with the right cuneus, the ability of discriminating outcomes increased from fair to good (AUC = 0.93).

## Discussion

The current study showed that patients with HFM, despite similar age, sex, baseline headache characteristics and psychiatric profiles, differing in neuroimaging features specifically over the posterior lobules of the bilateral cerebellar hemisphere had different 2-year headache outcomes.

In recent years, mounting evidence has shown that the cerebellum is not only involved in motor and coordinative functions, but is also involved in emotion, cognition, learning [30, 31]. In addition, cerebellum has been demonstrated to respond to noxious stimuli and involve pain processing [31–34], but the specific function of cerebellum during pain processing and its role in pain disorder is not clear. In migraine, cerebellar activation was demonstrated not only in trigeminal nociception but also during a migraine episode [35, 36]. Additionally, more silent ischemic lesions have been found over the cerebellum in patients with migraine with or without aura [37, 38]. These evidences link migraine pain and the consequences of repeated attacks with the cerebellum.

There were few studies reported volumetric changes of the cerebellum in patients with migraine, and the results varied with headache frequency. One study showed decreased cerebellar volume in patients with low frequency episodic migraine (mean monthly headache days = 4.7) [39]. Another study enrolled most patients with CM (episodic/chronic migraine: 8/46) showed increased GMV of the right cerebellar hemisphere, and the GMV increase was associated with a lower headache frequency and a shorter disease duration [40]. Our study in patients with HFM, in accordance with the latter one, also demonstrated a volume increase of the right cerebellar hemisphere, which correlated with higher disability (the MIDAS score). Further, our 2-year follow-up revealed an unfavorable outcome in those patients with greater cerebellar volume since they were less likely to have long-term headache remission. The above findings remained the same after adjusting for the covariates of baseline headache profiles. Therefore, the cerebellum not only involves the processing of migraine pain but also plays a role in migraine prognosis.

The role of cerebellum in migraine prognosis is also supported by other findings of the present study. A greater connectivity between the cerebellum and the cuneus, the calcarine cortex, and the temporal lobe, and a lesser connectivity between the cerebellum and the prefrontal cortex were associated with a poor headache prognosis. Earlier MRI studies in migraine also demonstrated functional connectivity change between the cerebellum and other brain structures. Functional connectivity was decreased between the cerebellum and the thalamus, occipital areas, and fusiformis gyrus, respectively, during trigeminal nociception in patients with migraine. The authors thus suggested a decreased inhibitory control of cerebellum on gating and nociceptive processing [40]. Another study showed increased resting functional connectivity of cerebellum with prefrontal cortex in patients with episodic migraine [39]. Taken together, through interacting with other brain structures, the cerebellum may modulate pain processing and pain persistence in patient with migraine.

Few neuroimaging studies have explored brain signatures of migraine prognosis. Our previous studies have shown that the volumes of the right hippocampus and the orbitofrontal cortex are associated with headache outcomes in patients with migraine and patients with CM with medication overuse, respectively [16, 41]. The current study showed the posterior lobe of cerebellum was associated with long-term migraine prognosis, specifically in patients with HFM without medication overuse. Although the mechanism of cerebellar involvement in headache prognosis is not clear, there were evidences that the larger cerebellar volume is associated with chronic widespread body pain [42]. Further, previous fMRI studies have shown the posterior lobes of cerebellum are activated during anticipation of pain [43]. Anticipation of pain lowers the behavior performance and increases pain intensity in patients with chronic pain [44]. More importantly, fear of pain, driven by the anticipation of pain, is a prognostic factor for chronic pain [45]. Whether this phenomenon and its association with cerebellum underlie the chronicity of migraine is of interest and needs to be further studied. Previous studies have shown varied cortical excitability in patients with migraine with different headache frequencies and outcomes [46, 47]. Further studies are required to determine if the cerebellum and its output contribute to the metaplasticity-like phenomenon at the cortical level and modulate cortical excitability in patients with migraine.

Some caveats and limitations should be addressed when interpreting the results of this study. First, to explore the brain signature related to outcome, we controlled for potential confounders that could possibly influence the GMV between the outcome groups. The GMV of the cerebellum remained significantly different between patients with different outcomes when we further controlled for disease duration and MIDAS score. However, there may be confounders that we did not identify in this study, such as musculoskeletal pain, that could potentially change brain GMV and headache prognosis. Second, the results of the current study were generated through VBM analysis. It should be borne in mind that some specific regions over the brainstem or limbic system may be too small for whole-brain analysis. The complex tissue pattern in these deep structures may also hamper the precision of VBM-based tissue segmentation. Third, the study was conducted with patients with either HFM or CM. HFM and CM are two clinical headache diagnoses arbitrarily separated by headache frequency of 10 and 15 days per month. Previous studies have shown similar symptom profiles, sociodemographic characteristics, and comorbidities between the two groups of patients [48]. These evidences support combining the two groups of patients when investigating their outcomes and neuroimage. Besides, the main study results were identical when we conducted the analysis only with patients with CM. Last, although we followed-up the clinical outcome of these patients at 2 years, we did not perform the second scan of brain MRI of these patients to see if the cerebellar volume changes are persistent. However, it should be noted that the patients with different outcomes had very different headache frequencies at 2-year follow-up, and their psychiatric conditions and the impact of their headaches may also vary with time. All these factors can cause unwanted confounding that hinders our identification of the brain signature predictive of headache outcome in patients with similar clinical profiles.

## Conclusion

Structural and connectivity changes of the cerebellum are associated with persistence or progression of headache frequency in patients with migraine with highly frequent attacks at baseline. Further studies are required to elucidate the role of the cerebellum in migraine chronification and investigate the potential role of the cerebellum in the treatment of refractory CM.

## Supplementary information

**Additional file 1.**Table 1**.** Demographics and clinical profile of patients with high frequency episodic migraine and chronic migraine.

**Additional file 2.** Table 2. Difference in GMV between patients with CM with different outcomes.

## Data Availability

Anonymized and statistical information of all the participants was made available to and shared only among qualified investigators.

## Abbreviations

HC: Healthy controls
HFM: High frequency migraine
CM: Chronic migraine
NRS: Numeric rating scale
TIV: Total intracranial volume
GMV: Gray matter volume
WM: White matter
VBM: Voxel based morphometry
SC: Structural covariance
MIDAS: Migraine Disability Assessment
HADS-A: The anxiety subscale of the Hospital Anxiety and Depression Scale
HADS-D: The depression subscale of the Hospital Anxiety and Depression Scale
