# Supplementary material for: The cerebellum is associated with 2-year prognosis in patients with high-frequency migraine
Source: J Headache Pain. 2020 Mar 18;21(1):29. doi: 10.1186/s10194-020-01096-4 (PMC7081533; doi:10.1186/s10194-020-01096-4)
Supplement: Supplementary file 1 — Additional file 1.Table 1. Demographics and clinical profile of patients with high frequency episodic migraine and chronic migraine. [file 10194_2020_1096_MOESM1_ESM.docx]

**Table 1. Demographics and clinical profile of patients with high frequency episodic migraine and chronic migraine**

|  | **High frequency episodic migraine**  **(n = 12)** | **Chronic migraine**  **(n = 44)** |
| --- | --- | --- |
| **Age** | 35.4 ± 11.5 | 41.6 ± 10.0 |
| **Sex (F/M)** | 8/4 | 35/9 |
| **Headache frequency-baseline (d/m)** | 10.9 ± 1.4^#^ | 21.4 ± 6.4 |
| **Disease duration (years)** | 13.6 ± 7.7 | 18.2 ± 12.0 |
| **Headache intensity (NRS 0-10)** | 7.0 ± 1.4 | 6.5 ± 1.8 |
| **MIDAS** | 32.8 ± 26.6 | 46.6 ± 52.8 |
| **HADS-A** | 7.6 ± 3.1 | 9.2 ± 4.5 |
| **HADS-D** | 4 ± 2.7^#^ | 7.5 ± 5.2 |
| **Headache frequency at 2-year follow-up (d/m)** | 6.5 ± 4.6 | 9.7 ± 10.3 |
| **Good outcome** | 8 (66.7%) | 29 (65.9%) |

NRS: numeric rating scale; MIDAS: Migraine Disability Assessment; HADS-A: the anxiety subscale of the Hospital Anxiety and Depression Scale; HADS-D: the depression subscale of the Hospital Anxiety and Depression Scale

^#^ denotes difference comparing patients with high frequency episodic migraine to chronic migraine, p < 0.05
