# Supplementary material for: The cerebellum is associated with 2-year prognosis in patients with high-frequency migraine
Source: J Headache Pain. 2020 Mar 18;21(1):29. doi: 10.1186/s10194-020-01096-4 (PMC7081533; doi:10.1186/s10194-020-01096-4)
Supplement: Supplementary file 2 — Additional file 2. Table 2. Difference in GMV between patients with CM with different outcomes. [file 10194_2020_1096_MOESM2_ESM.docx]

**Table 2. Difference in GMV between patients with CM with different outcomes**

| **MNI coordinates** | | | **Voxel size** | **Anatomical region** | **Local peak  T-value** |
| --- | --- | --- | --- | --- | --- |
| **x** | **y** | **z** |  |  |  |
| ***CM with good outcome < CM with poor outcome*** | | | | | |
| 14 | -14 | 1 | 572 | Right Thalamus | 3.47 |
| 33 | -54 | -60 | 4127 | Right Cerebellum VIIIa | 4.01 |
| -32 | -51 | -56 | 545 | Left Cerebellum VIIIa | 3.07 |
